# Discontinuation of long acting reversible contraceptive use and its determinants among women in Ethiopia: Systematic review and meta-analysis

**DOI:** 10.3389/fpubh.2022.979231

**Published:** 2022-12-06

**Authors:** Natnael Atnafu Gebeyehu, Kirubel Dagnaw Tegegne, Gebyaw Biset, Dagne Addisu Sewuyew, Biresaw Wassihun Alemu, Alemker Mola Yitayew

**Affiliations:** ^1^Department of Midwifery, College of Medicine and Health Science, Wolaita Sodo University, Sodo, Ethiopia; ^2^Department of Comprehensive Nursing, College of Medicine and Health Science, Wollo University, Dessie, Ethiopia; ^3^Department of Pediatrics and Child Health Nursing, College of Medicine and Health Science, Wollo University, Dessie, Ethiopia; ^4^Department of Midwifery, College of Medicine and Health Science, Debretabore University, Debre Tabor, Ethiopia; ^5^Department of Midwifery, College of Medicine and Health Science, Injbara University, Injbara, Ethiopia; ^6^School of Medicine, College of Medicine and Health Science, Wolaita Sodo University, Wolaita, Ethiopia

**Keywords:** contraceptive device, contraceptive agents, birth control, long acting, family planning, discontinuation, long acting contraceptive, Ethiopia

## Abstract

**Introduction:**

Contraception discontinuation is a major public health issue that leads to unwanted pregnancies and unsafe abortions. Therefore, this systematic review and meta-analysis aimed to estimate discontinuation of contraceptives and its determinants in Ethiopia.

**Methods:**

PubMed, Google Scholar, Scopus, Science Direct, and Addis Ababa University online library were searched. Data were extracted using Microsoft Excel and analyzed using STATA statistical software (v. 14). Publication bias was checked by forest plot, Begg's rank test, and Egger's regression test. To look for heterogeneity, I^2^ was computed, and an overall estimated analysis was carried out. Subgroup analysis was done by region, study setting, and publication. The pooled odds ratio for associated factors was also computed.

**Results:**

Out of 654 studies assessed, 20 met our criteria and were included in the study. The total number of study participants was 8,780. The pooled prevalence of discontinuation of long acting reversible contraceptive use was 36.94% (95% CI: 28.547–45.326). According to sub-group analysis, Amhara region (45%) and institution-based studies (47.9%) had the highest prevalence. The most common reason for contraceptive discontinuation was negative side effect (42.3%).

Women experienced side effects (AOR = 2.833:95% CI:2.005–4.003), didn't receive counseling on side effects (AOR = 2.417; 95% CI: 1.591–3.672), didn't appoint follow up (AOR = 2.820; 95% CI: 2.048–3.881), dissatisfied with the given service (AOR = 5.156; 95% CI: 3.644–7.296), and a desire to be pregnant (AOR = 2.366; 95% CI: 1.760–3.182) were predictors of discontinuation of contraceptives.

**Conclusion:**

In Ethiopia, the pooled prevalence of long acting contraceptive discontinuation was high. Side effects, not being informed about side effects, dissatisfaction with the provided service, no insertion follow-up, and a desire to become pregnant were all associated factors. Healthcare professionals should focus on the client's reproductive goals, proper management of side effects, counseling, and post-insertion visits.

**Systematic review registration:**

https://www.crd.york.ac.uk/prospero/display_record.php?ID=CRD42022347860, identifier CRD42022347860.

## Introduction

Reduced maternal mortality and universal access to reproductive health care are both improved by family planning (1, 2). As a result, it contributes to a 44% reduction in maternal mortality and a 21% reduction in deaths among children under the age of five (3). Long-acting reversible contraceptives include copper intrauterine devices and hormonal implants (4). In the world, 15% of women use long-acting reversible contraception (5). However, just 3% of women in Sub-Saharan Africa use long acting reversible contraception on a regular basis (6).

Even though the use of long-acting reversible contraception is improving, discontinuation is becoming to be a serious issue. Within a year of insertion, 20% of women in low-income countries discontinue using long acting reversible contraceptive methods (7). When women who “begin a contraceptive technique and cease it for any reason while still at risk of becoming pregnant,” this is known as contraceptive discontinuation (8). Contraception use, fertility, unexpected pregnancies, induced abortions, and miscarriages are all affected (9–11).

There has been a noticeable shift from an emphasis on “adopters” to a concern over “discontinuers” in the predictors of contraceptive ever-use across strategies, as per earlier studies (8, 12). Family planning programs would therefore be better off focusing on maintaining current users than on attracting new ones in order to avoid contraceptive discontinuation. Discontinuation can occur for a number of reasons, but the most common one is side effects that arise while in an emergency (8).

In Ethiopia, providing a long-acting form of family planning is a free but highly effective method of preventing unintended pregnancies (13). Cramping and changes in bleeding patterns are commonly reported as reasons for removal of the intrauterine uterine device and implant, with 6–17% of long-acting reversible contraceptive discontinuation occurring between 6 and 12 months (14).

Long-acting contraceptives are discontinued, which leads to unintended pregnancy, unwanted births, and abortion, all of which are detrimental to women's health. Every year, one-third of the 182 million pregnancies worldwide are unplanned, resulting in larger families, higher fertility, and social, economic, and physical problems (15). Evidence showed that after 3 months of use, 40% of women in Egypt, 51% in Kenya, 73% in Malawi, 56% in United Republic of Tanzania, and 47% in Zimbabwe were at risk of conception (10). By 3 years of use, 45% of intrauterine devices and 61% of implants had been discontinued in Ethiopia (16).

The primary goal of contraception is to avoid unwanted or untimely pregnancies (17). As a result, effective contraception might save 54 million unintended pregnancies, 79,000 maternal deaths, and one million baby deaths per year (18). In contrast, Ethiopia has one of the world's highest maternal death rates, with 412 per 100,000 live births (19). To address this, Ethiopia's Minister of Health outsourced long-acting family planning services to Health Extension Workers at the health post level (20).

Despite the fact that various primary studies have documented the proportion of long-acting contraceptive discontinuation in Ethiopia, there is no data at the country level. Therefore, the purpose of this systematic review and meta-analysis was to estimate the proportion of discontinued of long acting contraceptives and associated factors in Ethiopia. The results of this study will give family planning providers and other stakeholders the fundamental information they need to assist and manage women's discontinuation of long acting reversible contraception.

## Methods

### Reporting

This systematic review and meta-analysis study was conducted to determine cause, magnitude and its determinant of discontinuation of long acting reversible contraceptives in Ethiopia using the standard PRISMA checklist guideline (21) (Additional file 1). The review protocol was registered with the international prospective register of systematic review (PROSPERO) as number CRD42022347860.

### Search strategy

International online databases (Pub Med, Science Direct, Scopus, and Google Scholar) were used to search articles on the prevalence of discontinuation of long acting reversible contraceptives and determinant's. We also retrieved gray literature from Addis Ababa University's online research institutional repository. The search string was established using “AND” and “OR” Boolean operators. The following core search terms and phrases with Boolean operators were used to search related articles: ((((Discontinuation) OR (“Discontinuation” OR “Early removal”)) AND Long acting contraception) OR (“Long acting contraception” OR “Implants” OR “Implanon” OR “Jadelle” OR “Intrauterine contraceptive device” OR “Contraceptive” OR “Family planning” OR “Contraceptive device” OR “Contraceptive agent” OsR “Birth control device”)) AND Ethiopia. Search terms were based on PICO principles to retrieve relevant articles through the databases mentioned above. The search period covered studies conducted from 2010 to 2021 and published from 2015 to 2022 from April 1, 2022, to May 10, 2022.

### Outcome measurement

#### Long acting reversible contraceptive methods

Contraceptive methods which serve as 3–10 years but can be removed at any time; only implants and IUCD (22).

#### Discontinuation of long-acting reversible contraceptives

Initiating nd stopping long-acting reversible contraceptives before the end of the prescribed period due to a method difficulty (23).

### Inclusion and exclusion criteria

Only English language papers, including published and unpublished studies with full text available for search, and studies that took place in Ethiopia were included in this meta-analysis. This systematic review and meta-analysis excluded research that used duplicated sources, qualitative studies from developed nations, and articles without full text.

### Quality assessment

Two authors (NAG and KDT) independently appraised the standard of the studies using the Joanna Briggs Institute (JBI) standardized quality appraisal checklist (24). The disagreement raised during the quality assessment was resolved through a discussion led by the third author (BWA). Finally, the argument was solved and reached with an agreement. The critical analysis checklist has eight parameters with yes, no, unclear, and not applicable options. The parameters involve the following questions:

(1) Where were the criteria for inclusion in the sample clearly defined?
(2) Were the study subjects and, therefore, the setting described in detail?
(3) Was the exposure measured result validly and reliably?
(4) Were the main objective and standard criteria used to measure the event?
(5) Were confounding factors identified?
(6) Were strategies to affect confounding factors stated?
(7) Were the results measured indeed and dependably? and
(8) Was the statistical analysis suitable?. Studies were considered low risk when they scored 50% and above on the quality assessment indicators, as reported in Additional File 2.

### Risk of bias assessment

Two authors (NAG and BWA) independently assessed included studies for risk of bias through the bias assessment tool developed by Hoy et al. (25), consisting of ten items that assess four domains of bias and internal and external validity. Any disagreement raised during the risk of bias assessment was resolved through a discussion led by DS. Finally, the argument was solved and reached with an agreement. The first four items (items 1–4) evaluate the presence of selection bias, non-response bias, and external validity. The other six items (items 5–10) assess the presence of measuring the bias, analysis-related bias, and internal validity. Therefore, studies that received “yes” for eight or more of the ten questions were classified as “low risk of bias.” If studies that received “yes” for six to seven of the ten questions were classified as “moderate risk” whereas studies that received “yes” for five or fewer of the ten questions were classified as 'high risk' as reported in a Additional File 3.

### Data extraction

Microsoft Excel spreadsheet (2016) and STATA version 14 software were utilized for data extraction and analysis. Two authors (NAG and KDT) independently extracted all relevant data using a standardized Joanna Briggs Institute data extraction format. The disagreement raised during data extraction was resolved through a discussion led by the BA. Finally, the argument was solved and reached with an agreement. The data automation tool was not used due to this study's absence of the paper form (manual data). The name of the first author, year of publication, study region, study setting, study design, the prevalence of knowledge, attitude, practice, sample size, and quality of each paper was extracted.

### Data analysis

After extracting all relevant findings in a micro-soft excel spreadsheet, the data were exported to STATA software version 14 for analysis. The pooled prevalence of unintended pregnancy was computed using a 95% confidence interval. Publication bias was checked by funnel plot and more objectively through Begg and Egger's regression tests, with *P* < 0.05 indicating potential publication bias. The presence of between-study heterogeneity was checked by using the Cochrane Q statistic. This heterogeneity between studies was quantified using I^2^, in which a value of 0, 25, 50, and 75% represented no, low, medium, and high heterogeneity, respectively. A forest plot was used to visually assess the presence of heterogeneity, which presented at a high-level random-effect model was used for analysis to estimate the overall prevalence of knowledge, attitude, and practice of exclusive breastfeeding. Subgroup analysis was done by study setting, study design, study region, year of insertion and cause of discontinuation. Sensitivity analysis was executed to see the effect of a single study on the overall prevalence of the meta-analysis estimate. The findings of the study were presented in the form of text, tables, and figures.

## Results

### Search findings and study characteristics

**Figure 1:**
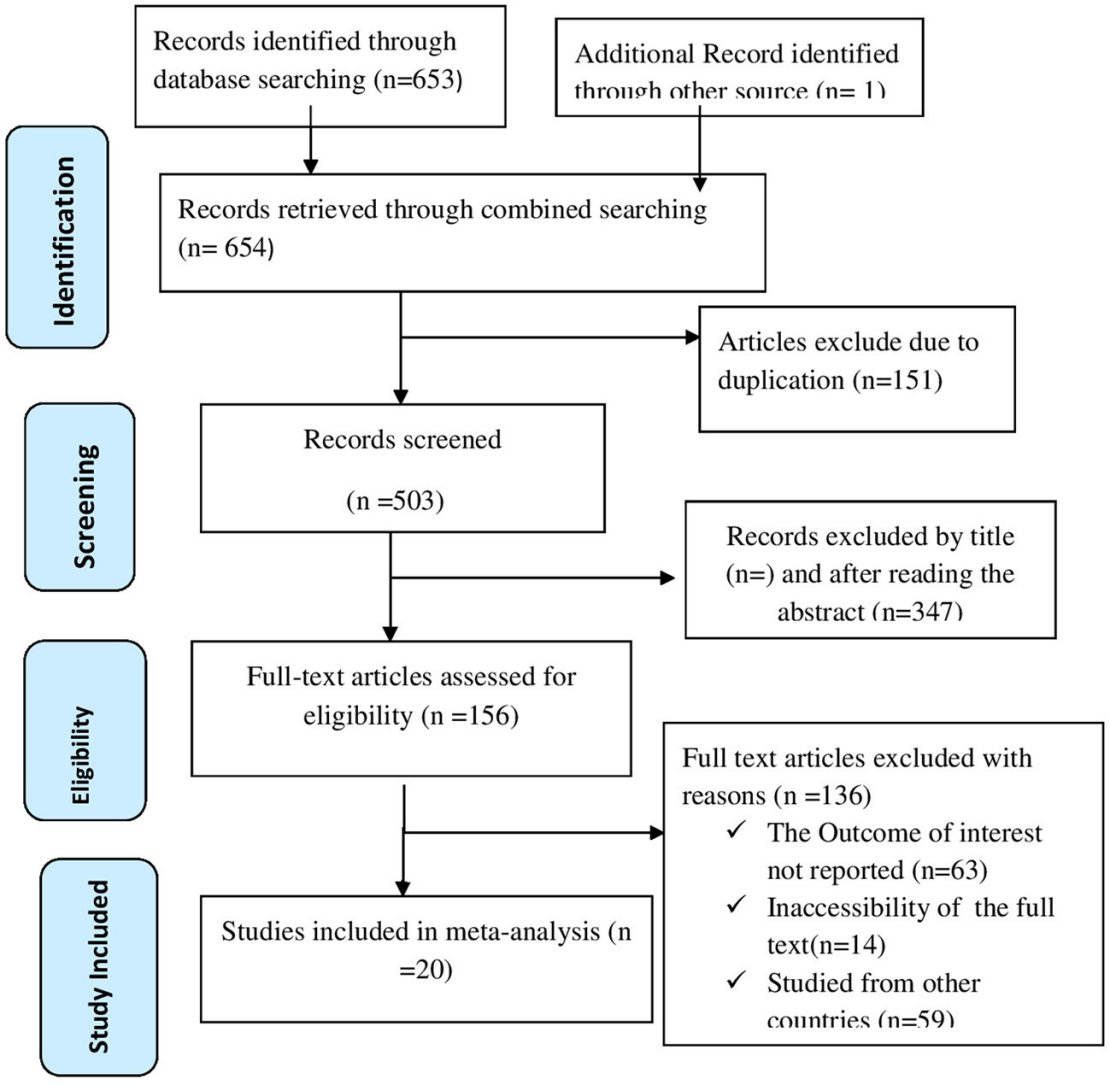
PRISMA flow chart displays the article selection process for discontinuation of long acting reversible contraceptives in Ethiopia.

Through online search engines such as PubMed, Scopus, Google Scholar, Science direct, and online research repository home, 654 articles were found using a search strategy on discontinuation of long acting reversible contraceptive methods in Ethiopia. There were 503 studies left after duplicates were deleted. After then, the remaining 503 studies were evaluated based on their entire titles and abstracts. As a result, 156 full-text articles were assessed for eligibility criteria, with 136 studies being excluded due to various factors. Finally, for this systematic review and meta-analysis study, 20 publications (23, 26–44) were used as presented by PRISMA diagram (Figure 1).

The cross-sectional study type was used in seventeen studies, whereas the retrospective cohort study approach was used in three. Both community and institutional studies were equally represented (10 studies in each). Nine studies conducted in Southern Nations Nationalists and Peoples Region (23, 27, 31, 32, 34–37, 39), five in Amhara (26, 28–30, 41), three in Tigray (40, 42, 43), and three in Oromia (33, 38, 44). The sample sizes ranged from 222 to 1050. The prevalence of discontinued of long acting contraceptive method ranged from 16 to 69.8. All of the studies were evaluated using the Joanna Briggs Institute (JBI) quality appraisal checklist, and all of them were found to be low risk (Table 1).

**Table 1:** Characteristics of the included studies in the systematic review and meta-analysis for the prevalence of discontinuation long acting reversible contraception in Ethiopia 2022.

**References**	**Region**	**Study setting**	**Study design**	**Sample size**	**Cause**	**Prevalence**	**quality**
Dagnew et al. (26)	Amhara	Institutional	cross-sectional	527	NR	37	Low-risk
Nageso and Gebretsadik (27)	SNNPR	Community	Cross-sectional	683	Side effect	23.4	Low- risk
Siyoum et al. (27)	Amhara	Community	Cross-sectional	314	Menstrual problem	40.5	Low- risk
Wondie et al. (29)	Amhara	Community	Cross-sectional	312	Side effect	16.7	Low -risk
Melkamu et al. (30)	Amhara	Institutional	Cross-sectional	499	Side effect	65	Low-risk
Weldekidan et al. (31)	SNNPR	Community	Cross-sectional	222	Menstrual problem	22.5	Low-risk
Gaenamo (32)	SNNPR	Institutional	Cohort	473	Desire of pregnancy	38.4	Low-risk
Abebe et al. (23)	SNNPR	Institutional	Cross-sectional	442	Menstrual problem	56.6	Low-risk
Obsu et al. (33)	Oromia	Commuity	Cross-sectional	360	Side effect	42	Low-risk
Mesha et al. (34)	SNNPR	Community	Cross-sectional	430	NR	34	Low-risk
Habte et al. (35)	SNNPR	Institutional	Cohort	502	Side effect	40.5	Low-risk
Amare and Reda (36)	SNNPR	institutional	Cross-sectional	351	Menstrual problem	49.3	Low-risk
Nega et al. (37)	SNNPR	Community	Cross-sectional	475	Side effect	23.2	Low-risk
Tesfaye et al. (38)	Oromia	Community	Cross-sectional	430	NR	19.3	Low-risk
Geja et al. (39)	SNNPR	Community	Cross-sectional	429	Desire of pregnancy	22.4	Low-risk
Tolesa et al. (44)	SNNPR	Institution	Cross-sectional	1,050	Side effect	69.8	Low-risk
Abraha et al. (40)	Tigray	Institutional	Cohort	413	Side effect	18.2	Low –risk
Gebrekidan et al. (42)	Tigray	Institutional	Cross-sectional	229	Desire of pregnancy	38	Low-risk
Birhane (43)	Tigray	Community	Cross-sectional	224	Health concern	16	Low-risk
Yilkal (41)	Amhara	Institutional	Cross-sectional	415	Side effect	66	Low-risk

###  Meta-analysis

#### Prevalence of discontinuation of long acting contraceptive methods in Ethiopia

**Figure 2:**
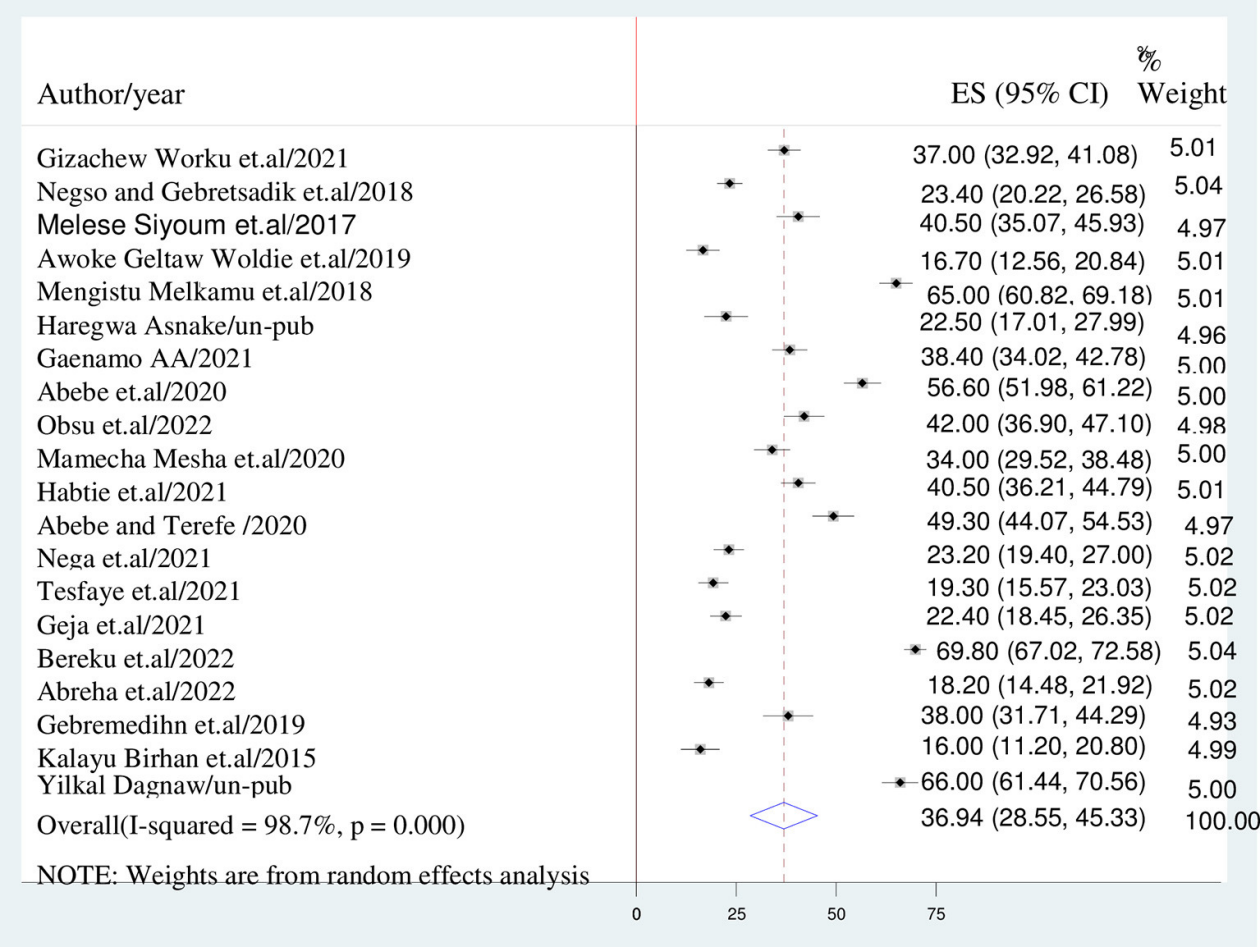
Forest plot displaying of discontinuation of long acting reversible contraceptives.

The overall burden of discontinuation of long acting contraceptives method is presented with a forest plot (Figure 2). Therefore, the national pooled estimate of discontinuation of long acting contraceptive methods in Ethiopia was 36.94% (95% CI: 28.547–45.326; I^2^ = 98.7%, *P* < 0.001).

### Sub-group analysis

The sub-group analysis was done based on study region, setting, cause of discontinuation, year of insertion and study design. The Amhara region had the highest rate of discontinuation of long-acting contraceptive methods (45%), whereas Oromia region had the lowest rate (30.6%). Institutional studies (47.9%) found a greater rate of long-acting contraceptive methods discontinuation than community-based research (25.9%). In terms of study design, cross-sectional studies (37.7%) revealed a higher rate of long-acting family planning method discontinuation than cohort studies (32.3%). On the other hand, side effects were cited as a main reason for the discontinuation of long-acting family planning methods (42.3%). Within three years, the highest rate of discontinuation of long-acting contraceptive methods (48.9%) was found (Table 2).

**Table 2:** The overall estimated of discontinuation of long acting reversible contraceptive in Ethiopia, 95% CI, and heterogeneity estimate with a *p*-value for sub-group analysis.

**Variables**	**Characteristics**	**Pooled estimate of LARCs 95% CI**	**I^2^ (*P*-value)**
Region	SNNPR	38.020% (25.995–50.045)	98.9% (0.000)
	Amhara	45.036% (26.098–63.975)	98.% (0.000)
	Tigray	36.937% (28.547–45.326)	94.2% (0.000)
	Oromia	30.581% (28.336–52.826)	98% (0.007)
Study setting	Institutional	47.897% (36.365–59.429)	98.7% (0.000)
	Community	25.891% (20.739–31.043)	93.2% (0.000)
Study design	Cross-sectional	37.748% (28.153–47.343)	98.9% (0.000)
	Cohort	32.330(17.731– 46.929)	97.3% (0.000)
Cause	Side effect	42.269% (27.869–56.669)	99.3% (0.000)
	Menstrual problem	40.529% (25.345–55.713)	6.8% (0.000)
	Desire for pregnancy	32.812% (21.491–44.132)	94.1% (0.000)
	Health concern	16% (28.547–45.326)	0.000(0.000)
Year of use	Within 1 year	29.518% (21.744–37.292)	97.6% (0.000)
	within 2 year	47.642% (28.547–45.326)	89.9% (0.000)
	Within 3 year	48.885% (39.691–58.079)	99.1% (0.000)

###  Leave–one-out-sensitivity analysis

A leave-one-out sensitivity analysis was carried out to detect the effect of each study on the overall prevalence of discontinuation of long acting contraceptive methods by excluding one study at a time. As a result, studies omitted at a time did not show a significant change on the overall prevalence of a discontinuation of long acting contraceptive method (Table 3).

**Table 3:** The pooled prevalence of discontinuation of long acting reversible contraceptives in Ethiopia when one study omitted from the analysis a step at a time 2022.

**Study omitted**	**Pooled estimate**	**95% CI**
Dagnew et al. (26)	36.934	28.075–45.793
Nageso and Gebretsadik (27)	37.655	28.888–46.421
Siyoum et al. (27)	36.751	28.004–45.497
Wondie et al. (29)	38.004	29.456–46.552
Melkamu et al. (30)	35.457	27.133–43.780
Weldekidan et al. (31)	37.691	29.030–46.352
Gaenamo (32)	36.860	28.034–45.686
Abebe et al. (23)	35.903	27.299–44.507
Obsu et al. (33)	36.671	7.911–45.432
Mesha et al. (34)	37.091	28.282–45.902
Habte et al. (35)	36.7492	27.919–45.579
Amare and Reda (36)	36.290	27.587–44.993
Nega et al. (37)	37.663	28.938–46.388
Tesfaye et al. (38)	37.869	29.254–46.485
Geja et al. (39)	37.705	29.005– 46.404
Tolesa et al. (44)	35.182	28.022–42.342
Abraha et al. (40)	37.928	29.350–46.506
Gebrekidan et al. (42)	36.882	28.169–45.594
Birhane (43)	38.036	29.482–46.590
Yilkal (41)	35.408	27.065–43.751

### Publication bias

**Figure 3:**
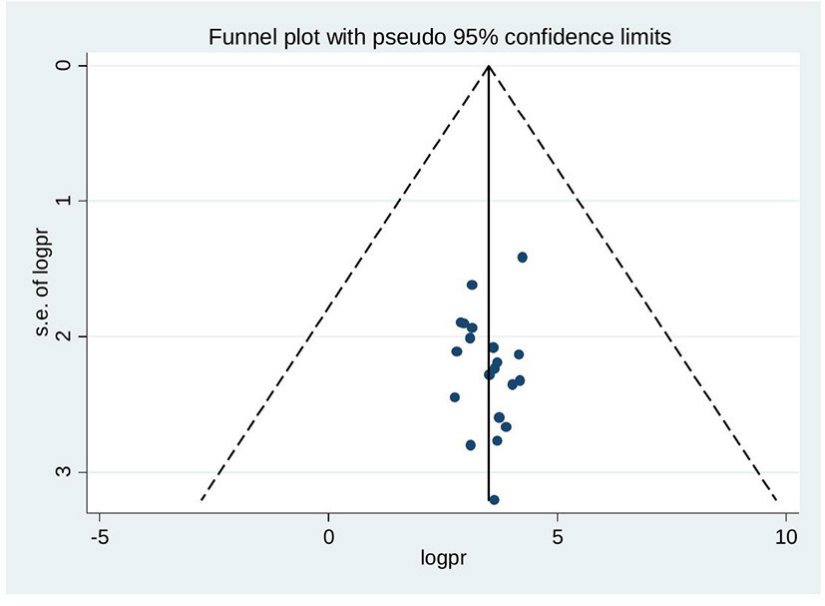
Funnel plot showing symmetrical distribution of studies indicating absence of publication bias.

The funnel plot was assessed for asymmetry distribution of discontinuation of long acting reversible contraceptives by visual inspection (Figure 3). The presence of publication bias was also assessed by Egger's regression test *p*-value of 0.875 and Begg's rank correlation test of *p*-value 0.230 with no evidence of publication bias.

### Factors associated with discontinuation of long acting reversible contraceptive methods in Ethiopia

**Figure 4:**
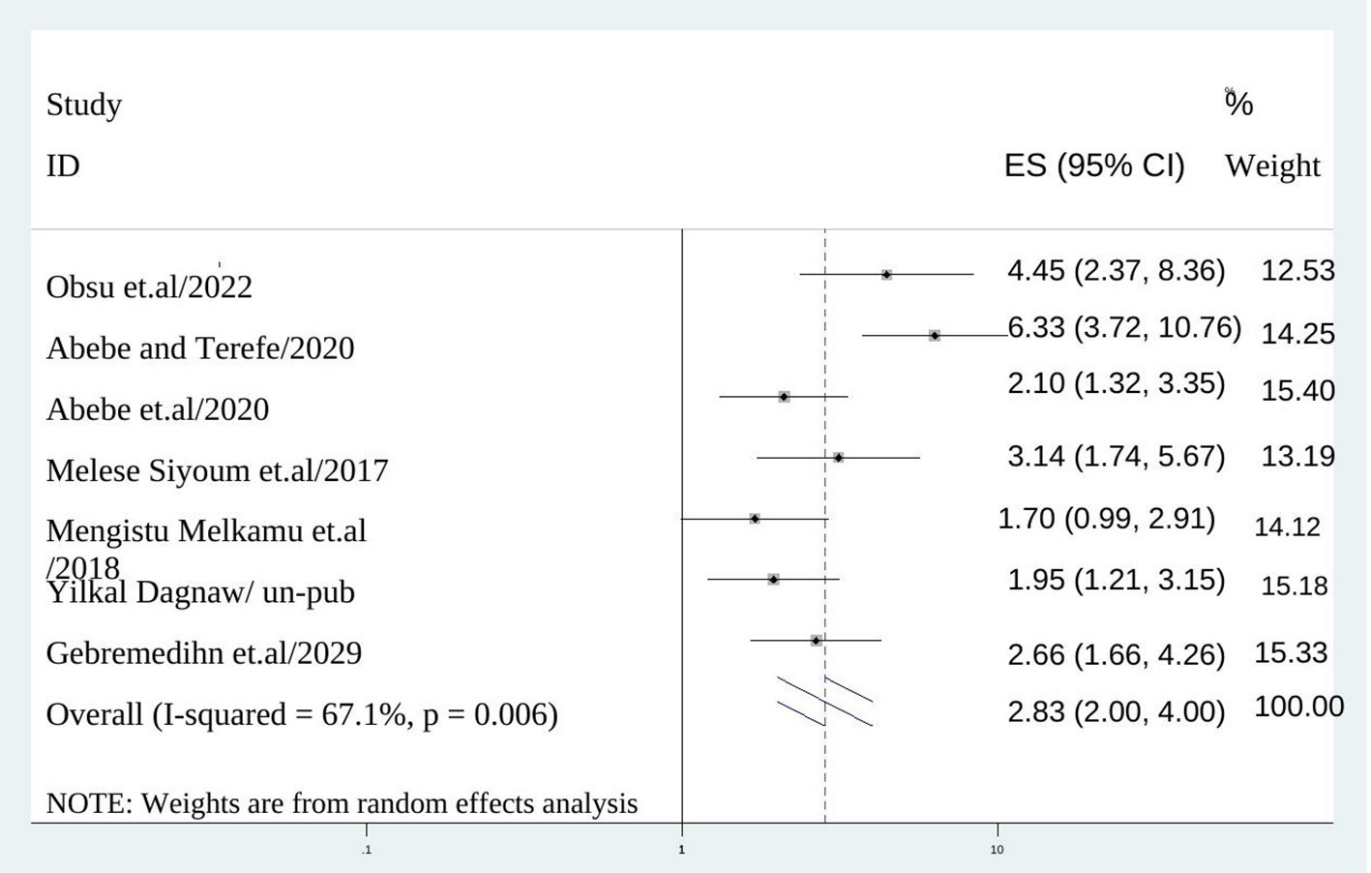
The association between experiencing side effects and discontinuation of long acting reversible contraception.

**Figure 5:**
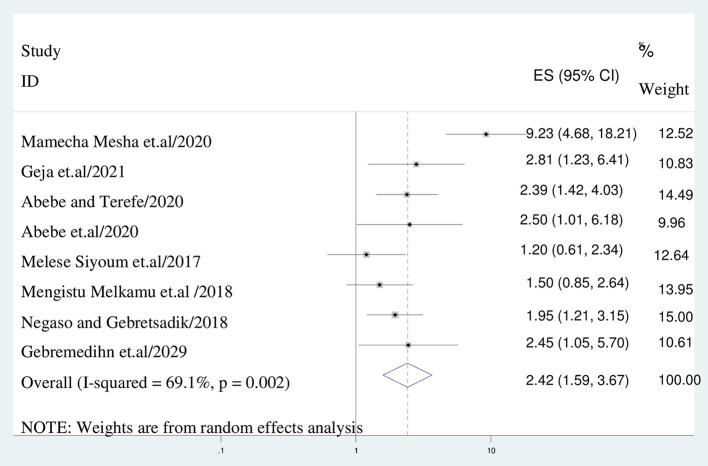
The association between lack of counseling on side effects and discontinuation of long acting reversible contraception.

In this study, women experienced side effect of long acting contraceptive methods had higher odds of family planning than their counter parts (AOR = 2.833:95% CI: 2.005–4.003), I^2^: 67.1%, *P* = 0.000) (Figure 4). In addition, the odds of having discontinuation of long acting contraceptives among mothers who didn't receive counseling on side effects was 2.4 times than their counter parts (AOR = 2.417; 95% CI: 1.591–3.672), I^2^: 69.1%, *P* = 0.000) (Figure 5).

**Figure 6:**
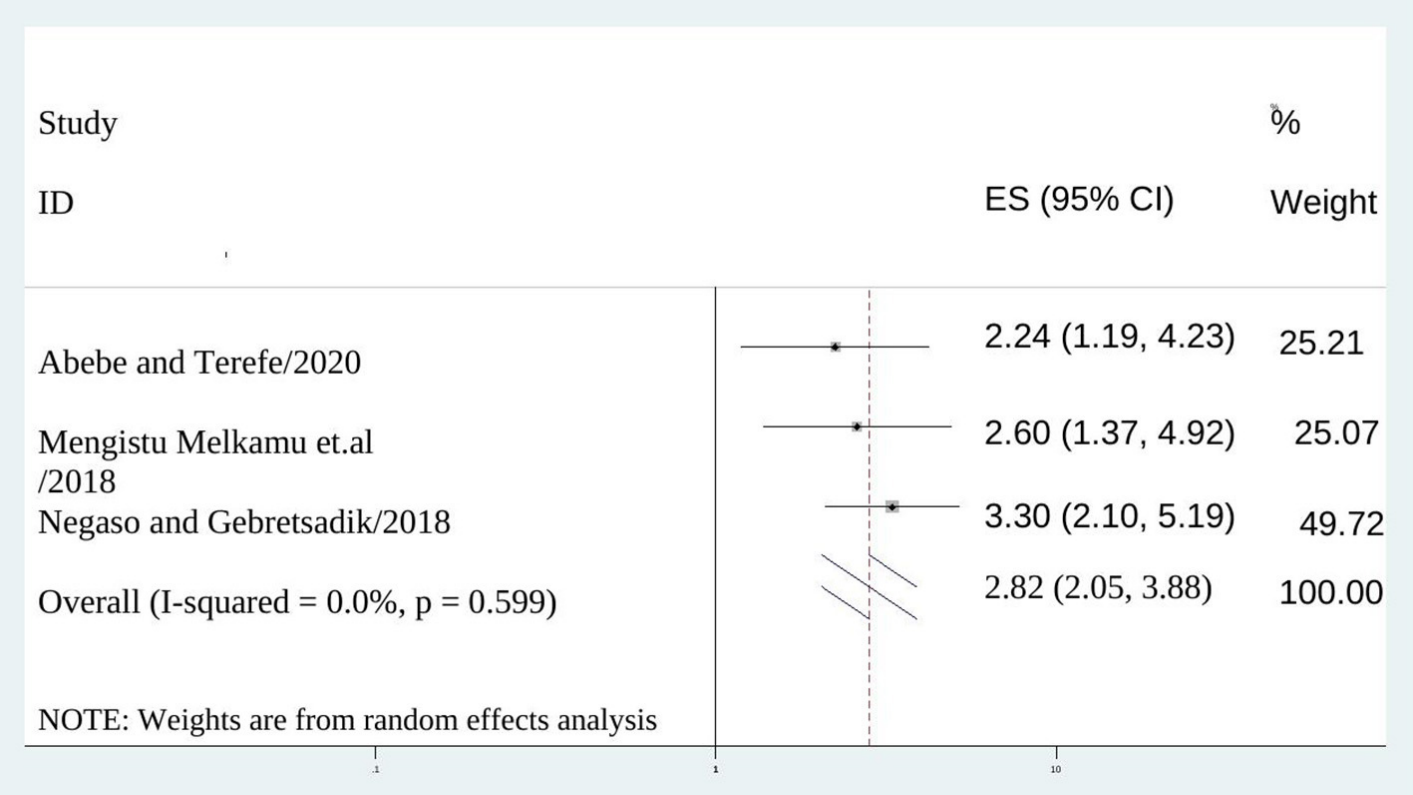
The association between not scheduled for follow up and discontinuation of long acting reversible contraception.

Women who had no follow up visit after post-insertion of long acting contraception were 2.8 (AOR = 2.820; 95% CI: 2.048–3.881), I^2^ = 0.0%, *P* = 0.000) times more likely to discontinue the method than mothers attending follow up visit (Figure 6).

**Figure 7:**
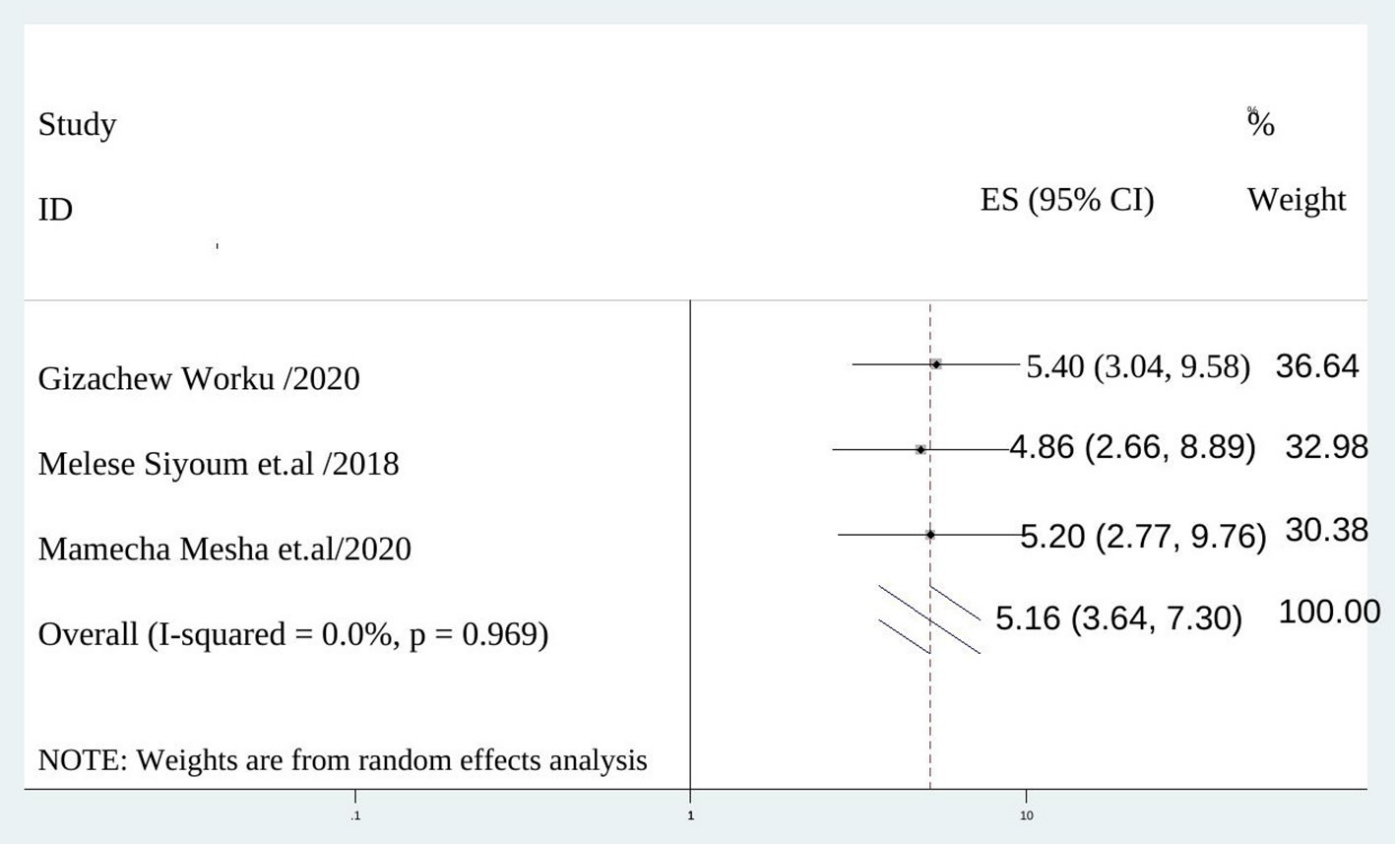
The association between not satisfied with provided service and discontinuation of long acting reversible contraception.

The chance of discontinuation of long acting contraceptive method was 5 times (AOR = 5.156; 95% CI :3.644–7.296), I^2^ = 0.0%, *P* = 0.000) among mothers didn't satisfy with services provided than those satisfied by service provision (Figure 7).

**Figure 8:**
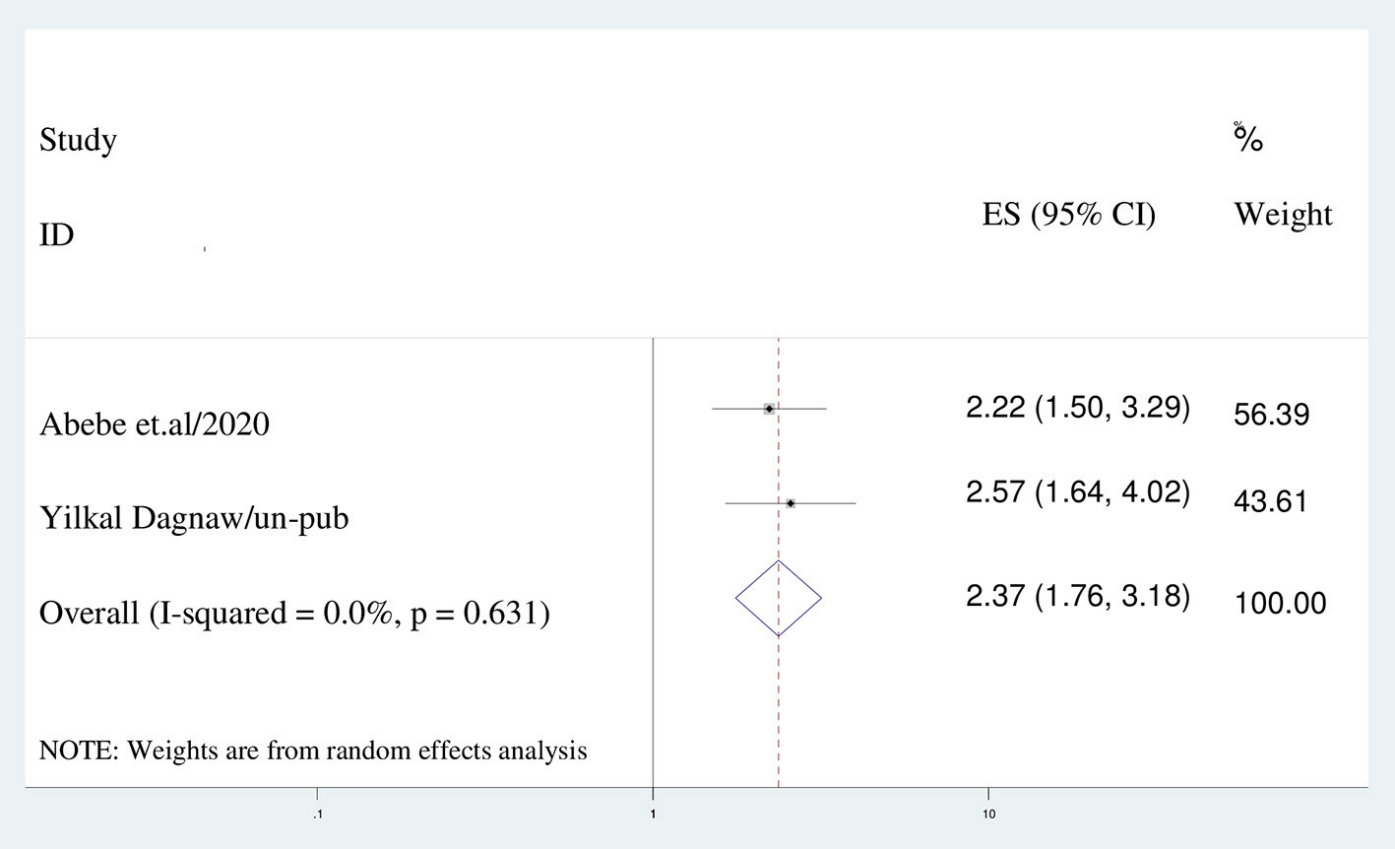
The association between a desire to be pregnant and discontinuation of long acting reversible contraception.

Women who had a desire of pregnancy were 2.4 (AOR = 2.366; 95% CI: 1.760–3.182), I^2^ = 0.0%, *P* = 0.000) times more likely to discontinue the method than women who had no desire of pregnancy (Figure 8).

## Discussion

The purpose of this systematic review and meta-analysis was to determine the magnitude of long-acting contraceptive discontinuation and its determinants among Ethiopian women. As a result, the overall prevalence of long-acting contraceptive discontinuation in this study was 36.94 % (95% CI: 28.547–45.326). The current study's findings are higher than those of studies conducted in Malaysia (22.86 %) (45), Nigeria (26.1%) (46), Egypt (26%) (47), a multicenter investigation in nine other countries (20%) (48), and meta-analysis study by Adal (7) (20 %). The possible explanation might be due to the difference in the composition of respondents as most of these studies are done by focusing on the rural population and time gap between studies. For example, the above mentioned meta-analysis done by Girum T. includes articles published from 1997 to 2017.

In contrast, the current study's findings are lower than those of research conducted in the Netherlands (47%) (49) and Australia (60%) (50). The time gap between the study and the attention given to the provision of long-acting contraception could be the cause of this discrepancy. This has been supported by the Ethiopian government's adoption of a health extension program to raise awareness and facilitate long-acting contraceptive availability at health post level in the community, which has a favorable impact on discontinuation rates (20, 51).

Sub-group analysis was done based on study region, setting, design, year of insertion and reason of discontinuation of long acting contraceptive methods. As a result, the Amhara region had a higher rate of contraceptive discontinuation (45%) than the Oromia region (35.6%). This could be due to regional socio-cultural and awareness differences. The discontinuation rates of institutional-based studies (47.9%) are greater than those of community-based studies (25.9%). It could be because mothers in institutional based research are sufficiently informed of their right to discontinue a method if they get pregnant or unsatisfied with a service.

The pooled prevalence of long-acting contraceptive discontinuation based on study design also showed significant difference, with 37.7% for cross-sectional studies and 32.3% for cohort studies, respectively. Indeed, cross-sectional studies determine prevalence rather than cohort studies, which identify and quantify risks. More evidence is needed, to the best of the researcher's knowledge, to justify this statistical discrepancy.

Side effects were indicated as a significant cause for discontinuing reversible long-acting contraceptives in the current study, which was supported by previous studies in Port Harcourt (7, 52). This could be due to women's intolerance of modest side effects from reversible long-acting contraceptive methods.

In this study, the proportion of discontinuation rate within the first year of insertion was 29.5 percent, which is higher than studies conducted in 20% of women in Adal (7), Nigeria (8%) (53), Spain (9%) (54), 14 developing countries (13.2%) (55), and Senegal (6.3%) (56). However, this result is lower than that of a research conducted in South Africa (967.3%) (57). The above discrepancy could be attributed to a lack of effective pre-insertion counseling about predicted side effects, sample size, socio-cultural differences, or the study's time gap. Another explanation could be that most health professionals in our country are reluctant to accept complaints of removal. The findings also revealed that there is no significant change in discontinuation rates between the second (47.6%) and third (48.8%) years of insertion, which is consistent with Bangladesh's (48%) (58).

In our study, women who had long-acting contraceptive side effects were 2.8 times more likely to stop using the method than those who did not (AOR = 2.833:95% CI: 2.005–4.003).This conclusion is supported by research from Kenya (59), Nigeria (60, 61), and Nepal (62). Women's intolerance of minor side effects and vaginal bleeding, which interferes with their sexual experience, could be a viable rationale. Another explanation could be that family planning professionals' lack of ability and experience in dealing with side effects leads to mothers' need to stop using the methods.

Women who were dissatisfied with the services they received were 5 times more likely to discontinue long-acting reversible contraceptives than mothers who were satisfied (AOR = 5.15; 95% CI: 3.644–7.296). This could be attributed to women who were uninterested in method selection, privacy, secrecy, communication competency, and explanation from service providers, all of which have an impact on service continuity.

Women who were not counseled about side effects of long acting reversible contraceptives were 2.4 times more likely to discontinue the method than their counterparts (AOR = 2.417; 95% CI: 1.591–3.672). This is in line with research from Nigeria (53) and Jordan (63). This is due to the fact that mothers who do not receive adequate side effect counseling may develop a negative attitude toward the method when they experience a side effect.

Women who did not schedule a follow-up appointment were 2.8 times more likely to discontinue than those who did (AOR = 2.820; 95% CI: 2.048–3.881). This is congruent with a study conducted in Port Harcourt (52). This means that during follow-up, women may receive more counseling time from care providers regarding managing side effects and receiving supportive treatment information.

Those who wanted to get pregnant were 2.4 times more likely to stop using long-acting contraceptives than women who didn't (AOR = 2.366; 95% CI: 1.760–3.182). This is consistent with a study done in Nigeria (61). Long-acting contraceptives must be discontinued when clients want to get pregnant (64).

To handle a large variance that occurred in between-study heterogeneity, a random-effect model was used in this research. We conducted leave-one-out sensitivity, and the results reveal that no single study had a substantial effect on the overall prevalence of contraception discontinuation.

We assessed the possible variability source *via* sub-group analysis using the study settings, regions, study design, cause of discontinuation, and year of insertion. The high heterogeneity might be due to differences in the sample populations, paper qualities, or socio-cultural, ethnic, and regional differences.

## Conclusion

In conclusion, the overall prevalence of long-acting contraceptive discontinuation in Ethiopia was high (36.9%). Furthermore, the pooled discontinuation rate differed by research region, setting, reason, design, and insertion year. Experienced side effects, not being advised about side effects, dissatisfaction with the provided service, no follow-up after insertion, and a desire to become pregnant were all variables that contributed to the discontinuation of long-acting reversible contraceptives, as per this study. The Amhara region of Ethiopia had the highest percentage of discontinuation. It is advisable to address discontinuation by recognizing the significance of side effects in counseling sessions, pregnancy intention, considering the sustainability of currently used contraceptives in view of Ethiopian women's philosophy, and effectively managing and counseling of side effects.

### Strength and limitation

This study has some limitations. First, there was no adequate similar study to compare the findings of the study. Second, articles were restricted to only being published in the English language and placed in Ethiopia. Third, some of the included studies were cross-sectional, which might affect the outcome variable because of other confounding factors. This research has also some strength. First, compressive electronic online international searching engines were used. Second, our review incorporated gray literature as part of the primary studies.

Third, discontinued long acting contraceptive predictors were discovered.

## Data availability statement

The original contributions presented in the study are included in the article/Supplementary material, further inquiries can be directed to the corresponding author/s.

## Author contributions

NG conceptualized the study. NG, KT, and BA contributed during data extraction and analysis. NG, GB, and AY wrote result interpretation. NG and KT prepared the first draft. NG, KT, DS, and BA contributed during the conceptualization, interpretation of results, and substantial revision. NG, KT, DS, GB, BA, and AY revised and finalized the final draft manuscript. All the authors read and approved the final version of the manuscript.

## Conflict of interest

The authors declare that the research was conducted in the absence of any commercial or financial relationships that could be construed as a potential conflict of interest.

## Publisher's note

All claims expressed in this article are solely those of the authors and do not necessarily represent those of their affiliated organizations, or those of the publisher, the editors and the reviewers. Any product that may be evaluated in this article, or claim that may be made by its manufacturer, is not guaranteed or endorsed by the publisher.
