# Supplementary material for: Discontinuation of long acting reversible contraceptive use and its determinants among women in Ethiopia: Systematic review and meta-analysis
Source: Front Public Health. 2022 Dec 6;10:979231. doi: 10.3389/fpubh.2022.979231 (PMC9763286; doi:10.3389/fpubh.2022.979231)
Supplement: Supplementary file 1 [file Table_1.DOCX]

Table2. Quality assessment for the included Studies using JBI standardized checklist

| Item | Clearly defined inclusion | Describe study setting and participant | Valid and reliable exposure measurement | Objective and standard criteria for measurement | Identified confounder | Strategies to deal with confounders | Valid and reliable outcome measurement | Appropriate statically analysis | No of ‘yes’ ‘ |
| --- | --- | --- | --- | --- | --- | --- | --- | --- | --- |
| Gizachew Worku et.al | Yes | Yes | No | Yes | Yes | No | Yes | Yes | 6/8=75 |
| Negaso & Gebretsadik | Yes | Yes | Yes | Yes | No | No | Yes | Yes | 6/8=75 |
| Melese Siyoum et.al | Yes | Yes | No | Yes | Yes | No | Yes | Yes | 6/8=75 |
| Awoke Geltaw Woldie et.al | Yes | Yes | No | Yes | Yes | Yes | Yes | Yes | 7/8=87.5 |
| Mengistu  Melkamu et.al | Yes | Yes | No | Yes | Yes | Yes | Yes | Yes | 7/8=87.5 |
| Haregwa Asnake | Yes | Yes | Yes | Yes | Yes | No | Yes | Yes | 7/8=87.5 |
| Abebe et.al | Yes | Yes | Yes | Yes | No | No | Yes | Yes | 6/8=75 |
| Obsu et.al | Yes | Yes | No | Yes | Yes | Yes | Yes | No | 6/8=75 |

| Mamecha Mesha et.al | Yes | Yes | Yes | Yes | No | | Yes | Yes | | No | 6/8=75 |
| --- | --- | --- | --- | --- | --- | --- | --- | --- | --- | --- | --- |
| Abebe and Terefe | Yes | yes | No | Yes | Yes | | Yes | Yes | | Yes | 7/8=87.5 |
| Nega et.al | Yes | Yes | Yes | No | Yes | | No | Yes | | Yes | 6/8=75 |
| Tesfaye et.al | Yes | Yes | Yes | Yes | Yes | | Yes | Yes | | No | 7/8=87.5 |
| Bereku et.al | Yes | Yes | Yes | Yes | Yes | | Yes | No | | Yes | 7/8=87.5 |
| Geja et.al | Yes | Yes | No | Yes | No | | Yes | Yes | | Yes | 6/8=75 |
| Gebremedihn et.al | Yes | Yes | Yes | Yes | Yes | | Yes | Yes | | No | 7 /8=87.5 |
| Yilkal Dagnaw et.al | Yes | Yes | No | Yes | Yes | | No | Yes | | Yes | 6/8=75 |
| Kalayu Birhan et.al | Yes | Yes | Yes | Yes | Yes | | Yes | Yes | | No | 7 /8=87.5 |
| **For cohort studies** | | | Gaenamo AA | | | Habtie et.al | | | Abreha et.al | | |
| Two groups are similar and recruited from the same population | | | Yes | | | Yes | | | Yes | | |
| Similar measurement of exposure both for exposed and  unexposed groups | | | Yes | | | Yes | | | Yes | | |
| Valid and reliable measurement of exposure | | | Yes | | | Yes | | | Yes | | |
| Identifying confounders | | | Yes | | | Yes | | | Yes | | |
| Strategies to deal with confounders | | | Yes | | | Yes | | | No | | |
| Groups are free of the outcomes at the beginning | | | No | | | No | | | Yes | | |
| Valid and reliable measurement of outcomes | | | Yes | | | No | | | No | | |
| Long enough follow-up time for the occurrence of outcomes | | | Yes | | | Yes | | | Yes | | |
| Complete follow-up time | | | No | | | No | | | Yes | | |
| Strategies to address lost follow-up | | | Yes | | | Yes | | | Yes | | |
| Percentage of yes (%) | | | 8/10=80% | | | 7/10=70% | | | 8/10=80% | | |
